# Professor V. Gudden

**Published:** 1886-07

**Authors:** 


### SOCIETY REPORTS.

---

CHICAGO MEDICAL SOCIETY.

*Stated Meeting, May 3, 1886.*—THE PRESIDENT, E. J. DOERING, M. D., in the chair.

DR. M. P. KOSSAKOWSKI exhibited a case of

DOUBLE HARE-LIP AND CLEFT PALATE IN AN INFANT.

He promised a subsequent report of the case.

DR. H. GRADLE read a paper on

PURE DRINKING WATER.

He said the only way of deciding the question whether the Chicago drinking water is ever deleterious to health is

